# Guillain Barre Syndrome associated with COVID-19 Infection: A Case Report

**DOI:** 10.31729/jnma.6710

**Published:** 2021-08-31

**Authors:** Sujan Shrestha, Niranjan KC, Niroj Hirachan

**Affiliations:** 1 Department of Medicine, Sumeru City Hospital, Lalitpur, Nepal; 2 Department of Intensive Care, Sumeru City Hospital, Lalitpur, Nepal; 3 Department of Anesthesia, Patan Academy of Health Sciences, Lalitpur, Nepal

**Keywords:** *case report*, *COVID-19*, *Guillain-Barré syndrome*

## Abstract

Coronavirus Disease has become a global pandemic after its emergence at the end of 2019 as a cluster of pneumonia. Apart from respiratory symptoms, neurologic complications are also common, mostly in hospitalized patients. More than 80 percent of patients have neurological symptoms during their disease course of which most common is encephalopathy. However, data on neurological complications like Guillain-Barre syndrome associated with coronavirus-2019 are scarce. Here, we report a case of a 64-years-old female patient with typical clinical and electrophysiological manifestations of Acute motor axonal neuropathy variant, who was reported positive with polymerase chain reaction for severe acute respiratory syndrome coronavirus-2, 13 days before the onset of acute bilateral weakness of extremities, areflexia, and normal sensory examination. Cerebrospinal fluid and electrophysiological examination were also suggestive. The neurological symptoms improved during treatment with immunoglobulins. Quick recognition of symptoms and diagnosis is important in the management of Guillain-Barre syndrome associated with coronavirus-2019.

## INTRODUCTION

Neurologic complications from Coronavirus disease-2019 are common in hospitalized patients with neurological symptoms in 80 percent at some point during their disease course.^1^ Neuro invasive and neurotrophic properties of COVID-19 have also been described in the literature. Neurological manifestations range from encephalopathy, myalgias, headache, dizziness, dysgeusia or anosmia, Stroke, movement disorders, motor, and sensory deficits, ataxia, seizures, and acute peripheral nerve disease. Several studies have also reported cases of neuromuscular disorders like Guillain-Barre syndrome (GBS) after COVID-19.^2^ GBS is a heterogeneous condition of acute immune-mediated polyneuropathies presenting as an acute, monophasic paralyzing illness provoked by a preceding infection.

## CASE REPORT

A 64-year-old female patient known case of diabetes mellitus with history of dry cough, throat discomfort, lethargy for 4 days was admitted to our hospital in May 2 2021. Owing to her Respiratory symptoms and positive contact history, Reverse-transcriptase-polymerase-chain-reaction (PCR) oropharyngeal test for COVID-19 was done which was positive. Her chest X-ray showed diffuse bilateral pulmonary opacities. She was managed with supplemental oxygen, antipyretics, Steroid, and Antibiotics. She also received remdesivir with an initial dose of 200 mg, followed by 100 mg daily for the next 4 days. She was discharged on day 11 of admission following improvement and negative RT-PCR report. She did not have any neurological deficits during the time of discharge.

She was readmitted two days later with an acute onset symmetric weakness of extremities (Medical Research Council/MRC/scale was 3/5 in lower extremities and 4/5 in upper extremities), Areflexia (absent ankle and knee reflex in bilateral lower limb) with intact sensory examination and intact cranial nerves. She had normal bowel and bladder function. Her weakness began 13 days following a positive reverse-transcriptase-polymerase-chain-reaction (PCR) oropharyngeal test for COVID-19 and 17 days following her first symptoms. The general physical examination showed severe dehydration, although she was afebrile. Meningeal irritation signs and upper motor neuron disorder signs were negative. The laboratory examination results were as follows: serum glucose 654 mg/dL; urea 188 mg/dL; creatinine 2.3 mg/dL; total bilirubin1.6 mg/dl: direct bilirubin 0.5mg/dl; alanine aminotransferase 71 U/L; aspartate aminotransferase 55 U/L; ALP 103 IU/ml; sodium 155 mmol/L; potassium 5.8 mmol/L; white blood cell count 27,400 cells per microliter (neutrophils = 90%; lymphocytes = 7%); CPK-MB 151 U/L; haemoglobin 15.7 g/dL, positive for ketone in complete urinalysis and arterial blood gas showed high anion gap metabolic acidosis. Diagnosis of Diabetic Ketoacidosis was made and managed accordingly.

Her lower limb Weakness progressed to Medical Research Council/MRC/scale of 2/5 over next 7 days, although her upper limb weakness was static. The modified Erasmus Guillain-Barre Syndrome outcome score (mEGOS) was 5 at day 7 of hospitalization pointing to a favourable outcome. Cerebrospinal fluid (CSF) assessment done on day 8 showed an albumin-cytologic dissociation with increased protein level(64g/L) and normal cell count (3 cells/mm3). CSF SARS-Cov-2 RNA was negative. Later Standard laboratory tests (complete blood count, CRP, serum glucose, creatinine, sodium and potassium level, TSH, creatine kinase, and urine test) and special blood tests (HIV, serum vitamin B12-level, and serum protein) were also within the normal range. Magnetic resonance imaging (MRI) of the brain and cervical spine were normal.

**Figure 1:**
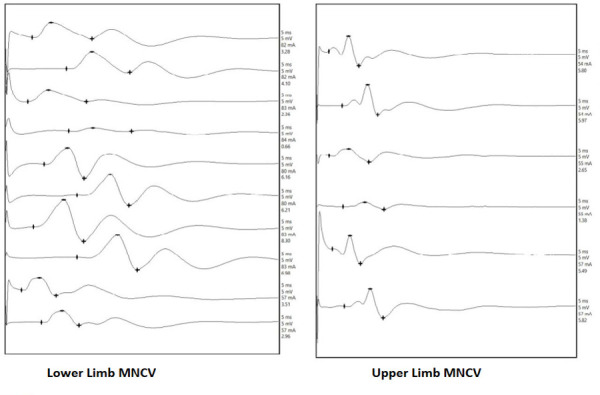
Motor conduction study with normal distal latency and decreased amplitude of CMAP of motor nerves tested in upper and lower limb predominantly affecting lower limb.

Lower extremities weakness was further progressive and clinical examination on 13^th^ day of readmission, showed power of 1/5 on both lower limbs. Electrophysiological study was performed using a NeuroStimEMG device on day 14. The electrophysiological evaluation showed Motor conduction study with normal distal latency and decreased amplitude of CMAP of motor nerves tested in upper and lower limb predominantly affecting lower limb (Figure 1) (Table 1). Absent F-Wave and H-Wave in motor nerves tested in lower limbs. Sensory conduction study sowed normal SNAP of sensory nerves tested in both upper and lower limbs (Table 2). NCV features were suggestive of early axonal motor neuropathy predominantly affecting lower limbs with normal sensory studies, likely due to AMAN variant of GBS.

**Figure 2:**
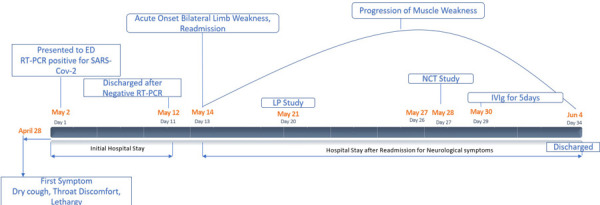
Timeline of clinical events and diagnostic investigations. COVID-19 coronavirus disease-2019, GBS Guillain-Barre syndrome, LP lumbar puncture, IVIg intravenous immunoglobulin.

She received intravenous immunoglobulin at the dose of 0.4 g/kg/day staring from day 16 over the course of 5 day. At the time of discharge on day 21, her Medical Research Council/MRC/scale was 3/5 in proximal and distal lower extremities, and 4/5 in upper extremities. Timeline of all clinical events from admission to discharge has been shown (Figure 2).

**Table 1:** Motor Nerve conduction study

Nerve	Latency (ms)		Amplitude (mV)			Duration (ms)			Dist. (mm)	NCV (m/s)	F-Min (ms)	F-Max (ms)
	D	P	D	P	%Dec	D	P	%Inc				
Rt. CPN	4.81	11.06	3.28	4.10	25.00	10.87	11.56	6.35	300.00	48.00	25.00	30.00
Lt. CPN	4.00	11.44	2.36	0.66	72.03	10.69	11.50	7.58	310.00	41.67	25.00	30.00
Rt. PTN	7.00	13.00	6.16	6.21	0.81	7.25	9.44	30.21	270.00	45.00	52.00	52.25
Lt. PTN	5.06	13.00	8.30	6.98	15.90	9.12	10.94	19.96	300.00	37.78	52.75	52.62
Rt. Median	2.87	6.50	3.51	2.96	18.67	6.31	6.87	8.87	210.00	57.85	-	-
Rt. Ulnar	2.31	4.87	5.80	5.97	2.93	5.75	6.81	18.43	230.00	89.84	-	-
Lt Median	2.37	5.06	2.65	1.38	47.92	7.56	7.87	4.10	210.00	78.07	-	-
Lt. Ulnar	3.00	5.25	5.49	5.82	6.01	5.37	7.50	39.66	210.00	93.33	-	-

**Table 2:** Sensory conduction study with normal SNAP of sensory nerves tested in both upper and lower limbs.

Nerve	Latency (ms)	Amplitude (μV)	Distance (mm)	NCV (m/s)
Rt. Sural	3.00	10.42	140.00	46.67
Lt. Sural	3.23	10.47	140.00	43.34
Rt. Median	3.00	26.67	160.00	53.33
Rt. Ulnar	1.80	15.33	130.00	72.22
Lt. Median	2.44	21.53	140.00	57.38
Lt. Ulnar	1.99	17.49	130.00	65.33

## DISCUSSION

GBS is a heterogeneous condition of acute immune-mediated polyneuropathies presenting as an acute, monophasic paralyzing illness provoked by a preceding respiratory or Gastrointestinal tract infection in approximately two-thirds of patients.^3^ Guillain-Barre syndrome (GBS) occurs worldwide with an overall incidence of 1 to 2 cases per 100,000 per year.^4^ Antecedent infections with Campylobacter jejuni infection are the most commonly identified. GBS also occurs in association with human immunodeficiency virus (HIV) infection, following influenza-like illnesses, cytomegalovirus and Epstein-Barr virus infections, and less commonly with a varicella-zoster virus; herpes simplex virus; hepatitis A, B, C, and E; chikungunya virus; and the bacteria Haemophilus influenzae, Escherichia coli, and Mycoplasma pneumonia.^5^ Older coronavirus-types (SARS-severe acute respiratory syndrome and MERS-middle east respiratory syndrome) and Zika virus have been associated with GBS.^4^ Several reports have emerged as GBS associated with COVID-19.^2^

Antecedent infection evokes an immune response, which in turn cross-reacts with peripheral nerve components because of the sharing of cross-reactive epitopes (molecular mimicry).^4^ Acute inflammatory demyelinating polyradiculoneuropathy (AIDP) is the most common form of GBS in the United States and Europe. In AIDP and Miller Fischer variant, a focal inflammatory response develops against myelin-producing Schwann cells or peripheral myelin. Acute motor axonal neuropathy (AMAN) is the primary axonal form of GBS which occurs frequently in ASIA particularly in young people.^6^ In the motor and motor-sensory variants, the axon is affected without an inflammatory response, and the primary immune process is directed at the nodes of Ranvier.

SARS-CoV-2 is capable of causing severe systemic inflammation consistent with a cytokine release syndrome with an increased level of cytokines as TNF-alpha, Interleukin-6 (IL-6). Based on the timing of symptoms relative to initial symptoms of COVID-19 infection, GBS occurs mostly as a para infectious and in few cases as a postinfectious complication. But exact pathogenies and casual relationships of GBS and COVID-19 have not been established.

GBS predominantly AIDP presents with progressive, fairly symmetric muscle weakness accompanied by absent or depressed deep tendon reflexes, usually present a few days to a week after onset of symptoms. The weakness can vary from mild difficulty with walking to nearly complete paralysis of all extremity, facial, respiratory, and bulbar muscles.^4^ Deep tendon reflexes are occasionally preserved in patients with AMAN. Sensory nerves are not affected.^7^ Our review of the literature revealed, all patients had a fever and respiratory symptoms 5 to10 days before the onset of the neurological symptoms, one of them had an ongoing fever.^2^ The clinical findings of our patient were consistent with GBS.

lumbar puncture in patients with GBS reveals an elevated cerebrospinal fluid (CSF) protein with a normal white blood cell count, also known as albumin-cytologic dissociation.^4^ Electrodiagnostic studies (nerve conduction studies) may show evidence of an acute polyneuropathy with predominantly demyelinating features in acute inflammatory demyelinating polyradiculoneuropathy (AIDP) like prolonged or absent F waves and absent H reflexes, reflecting demyelination at the level of the nerve roots, Increased distal latencies and conduction blocks with temporal dispersion of motor responses follow Significant slowing of nerve conduction velocities usually in the third or fourth week and reduced recruitment in Needle electromyography (EMG) of weak muscles. There are predominantly axonal features (low distal motor and/or sensory amplitudes) in acute motor axonal neuropathy (AMAN) and acute motor and sensory axonal neuropathy (AMSAN).^8^ Antibodies test against GQ1b and glycolipids are not routinely done. Similarly, Spinal MRI if done may reveal thickening and enhancement of the intrathecal spinal nerve roots and cauda equina. In reported cases, COVID-19 is associated with AIDP, AMN, and Miller Fischer variant. Antiganglioside antibodies were absent in most cases of GBS associated with COVID-19.^2^ The LP and electrophysiological findings of our patient were consistent with the AMAN variant of GBS.

Quick recognition of symptoms and diagnosis is important in the management of these patients. Supportive care for patients with Guillain-Barre syndrome (GBS) is extremely important due to the associated risk of respiratory failure and autonomic dysfunction with potentially severe cardiovascular involvement.^9^ We propose that close attention to neurologic complications including GBS should be given in COVID-19 patients. Treatment with plasma exchange or intravenous immune globulin (IVIG) is otherwise indicated for most patients with GBS because these treatments accelerate recovery.^10^ However, their potential benefit in the setting of COVID-19 should be properly studied.
